# Surveying the molecular landscape of pediatric brain tumors via spatially resolved transcriptomics

**DOI:** 10.1093/bib/bbaf631.031

**Published:** 2025-12-12

**Authors:** Javier Escudero Morlanes, Timo-Pekka Lehto, Ludvig Larsson, Leire Alonso Galicia, Annelie Mollbrink, Alia Shamikh, Elisa Basmaci, Gabriela Prochazka, Teresita Diaz De Ståhl, Johanna Sandgren, Fulya Taylan, Bianca Tesi, Ann Nordgren, Andrew Erickson, Alastair D Lamb, Klas Blomgren, Monica Nistér, Joakim Lundeberg, Reza Mirzazadeh, Linda Kvastad

**Affiliations:** Department of Gene Technology, KTH Royal Institute of Technology, Science for Life Laboratory, Solna, Sweden; Department of Urology, University of Helsinki and Helsinki University Hospital, Helsinki, Finland; Department of Pathology, University of Helsinki and Helsinki University Hospital, Helsinki, Finland; Research Program in Systems Oncology, Faculty of Medicine, University of Helsinki, Helsinki, Finland; Department of Gene Technology, KTH Royal Institute of Technology, Science for Life Laboratory, Solna, Sweden; Department of Gene Technology, KTH Royal Institute of Technology, Science for Life Laboratory, Solna, Sweden; Department of Gene Technology, KTH Royal Institute of Technology, Science for Life Laboratory, Solna, Sweden; Department of Oncology-Pathology, Karolinska Institutet, Stockholm, Sweden; Clinical Pathology and Cancer Diagnostics, Karolinska University Hospital, Stockholm, Sweden; Department of Oncology-Pathology, Karolinska Institutet, Stockholm, Sweden; Clinical Pathology and Cancer Diagnostics, Karolinska University Hospital, Stockholm, Sweden; Department of Oncology-Pathology, Karolinska Institutet, Stockholm, Sweden; Clinical Pathology and Cancer Diagnostics, Karolinska University Hospital, Stockholm, Sweden; Department of Oncology-Pathology, Karolinska Institutet, Stockholm, Sweden; Clinical Pathology and Cancer Diagnostics, Karolinska University Hospital, Stockholm, Sweden; Department of Oncology-Pathology, Karolinska Institutet, Stockholm, Sweden; Clinical Pathology and Cancer Diagnostics, Karolinska University Hospital, Stockholm, Sweden; Department of Molecular Medicine and Surgery, Karolinska Institutet, Stockholm, Sweden; Clinical Genetics and Genomics, Karolinska University Hospital, Solna, Sweden; Department of Clinical Genetics and Genomics, Karolinska University Hospital, Stockholm, Sweden; Department of Molecular Medicine and Surgery, Karolinska Institutet, Stockholm, Sweden; Department of Clinical Genetics and Genomics, Karolinska University Hospital, Stockholm, Sweden; Department of Molecular Medicine and Surgery, Karolinska Institutet, Stockholm, Sweden; Systems Oncology, Faculty of Medicine, University of Helsinki, Helsinki, Finland; Nuffield Department of Surgical Sciences, University of Oxford, United Kingdom; Systems Oncology, Faculty of Medicine, University of Helsinki, Helsinki, Finland; Nuffield Department of Surgical Sciences, University of Oxford, United Kingdom; Department of Urology, Oxford University Hospitals NHS Foundation Trust, United Kingdom; Department of Women’s and Children’s Health, Karolinska Institutet, Stockholm, Sweden; Department of Paediatric Oncology, Karolinska University Hospital, Stockholm, Sweden; Department of Oncology-Pathology, Karolinska Institutet, Stockholm, Sweden; Clinical Pathology and Cancer Diagnostics, Karolinska University Hospital, Stockholm, Sweden; Department of Gene Technology, KTH Royal Institute of Technology, Science for Life Laboratory, Solna, Sweden; Department of Gene Technology, KTH Royal Institute of Technology, Science for Life Laboratory, Solna, Sweden; Department of Gene Technology, KTH Royal Institute of Technology, Science for Life Laboratory, Solna, Sweden; Current affiliation: Department of Cell and Molecular Biology, Karolinska Institute, Solna, Sweden

## Abstract

Due to their intrinsic heterogeneity and plasticity, pediatric brain tumors present highly complex clinical challenges. To provide insight into the molecular scene of these tumors, we generated spatial transcriptomics data from 19 distinct patients, 7 of which suffered a relapse of the disease. In this cohort, spanning 59 tissue sections across 8 diagnoses and 4 tumor grades, we recovered diagnosis-specific gene expression patterns that could be linked to processes characteristic of developmental stages. We also identified between 2 to 4 spatial archetypal niches per section, which could then be related to 11 main biological themes, and to distinct celltype-like transcriptomic signatures. For example, we noted strong spatial correlations between developmental archetypes and oligodendrocyte lineage signal in pilocytic astrocytoma. Lastly, we utilised spatially inferred copy-number variants for the profiling of relapse-associated tentative tumor clones. These clones were then related via spatial geographical analysis to regions with strong blood vessel signatures. Overall, these results provide vital details into the progression and maintenance of pediatric brain tumors, offering novel edges to be exploited in personalized medicine.

